# The Promising IgSF11 Immune Checkpoint Is Highly Expressed in Advanced Human Gliomas and Associates to Poor Prognosis

**DOI:** 10.3389/fonc.2020.608609

**Published:** 2021-02-02

**Authors:** Amina Ghouzlani, Soumaya Rafii, Mehdi Karkouri, Abdelhakim Lakhdar, Abdallah Badou

**Affiliations:** ^1^ Cellular and Molecular Pathology Laboratory, Faculty of Medicine and Pharmacy, Hassan II University, Casablanca, Morocco; ^2^ Department of Pathology, CHU Ibn Rochd, Casablanca, Morocco; ^3^ Department of Neurosurgery, UHC Ibn Rochd, Casablanca, Morocco; ^4^ Laboratory of Research on Neurologic, Neurosensorial Diseases and Handicap, Faculty of Medicine and Pharmacy, Hassan II University, Casablanca, Morocco

**Keywords:** IgSF11, PDL-1, VISTA, glioma, glioblastoma, immune checkpoint

## Abstract

Glioma is the most prevalent primary brain tumor. Immune checkpoint blockade has made a great stride in mending patient’s clinical outcome for multiple types of cancers. However, PD-1, CTLA-4, or VEGF blockade exhibited only poor outcome in glioma patients. This study aimed to explore the expression and role of IgSF11, an emerging immune checkpoint and a ligand of VISTA, in human gliomas. IgSF11 mRNA expression was assessed in human glioma patients at different grades using 2 independent cohorts, a set of 52 Moroccan samples, including 20 glioma tissues, 22 PBMC samples taken before and 10 PBMC samples taken after surgery; and a series of 667 patients from TCGA. In parallel, immunohistochemistry was performed to evaluate IgSF11 protein staining. *IgSF11* gene expression was significantly upregulated in high grade glioma tissues, compared to low grade. IgSF11 protein also showed a significant expression in low and high-grade gliomas. Interestingly, IgSF11 expression seemed to correlate positively with other critical immune checkpoints such as PD1, PDL-1, VISTA, and surprisingly negatively with CTLA-4. Although, T cell markers appeared higher in advanced gliomas, T cell-produced pro-inflammatory genes showed similar expression levels, highly likely because of the potent immunosuppressive microenvironment. Indeed, increased expression of IgSF11 in advanced human gliomas associated with a poor overall survival. Our data strongly suggest that IgSF11 is an immune checkpoint, which is upregulated in advanced human gliomas and contributes to the immunosuppressive state resulting in a poor clinical outcome in glioma patients. IgSF11 could be considered as a possible promising therapeutic target in advanced human gliomas.

## Introduction

Immunotherapy can reach long-lasting tumor remission with minimal adverse effects through manipulating the immune system (1). Among the most emerging strategies to fight cancer progression and activating therapeutic antitumor immunity is the blockade of immune checkpoints (2). Gliomas represent the most common primary tumor of the brain that appears from its intrinsic constituent cells (3, 4). In addition to its high frequency among all types of central nervous system tumors, glioma exhibits the most aggressive and lethal type of cancers (5, 6), glioblastoma (GBM) or glioma of grade IV, according to the World Health Organization (WHO) classification of tumors of the central nervous system (CNS) (7). Despite conventional therapies such as surgical resection, concomitant chemoradiotherapy, and/or adjuvant chemotherapy, the prognosis of patients with GBM remains poor (8). In the last two decades, a set of data has displayed dramatic success of immune checkpoint blockade targeting, the PD1- PDL1 axis and/or cytotoxic T lymphocyte associated antigen 4 (CTLA4), in different types of solid tumors (9–13). On the other hand, a Phase III trial comparing nivolumab (anti-PD-1 blocking Ab) to bevacizumab in patients with recurrent GBM failed to substantiate the benefit of nivolumab, which conferred a similar median overall survival (mOS, 9.8 vs. 10.0 months) (14). Previous studies have shown that the combination of anti-PD-1 and anti-CTLA-4 blocking Abs also does not improve the overall survival (15). Therefore, no obvious benefit of neoadjuvant nivolumab was obtained with resectable GBM, with a median overall survival of just 7.3 months (14).

The immunoglobulin superfamily 11 gene (IgSF11), also known as V-Set and Immunoglobulin domain containing 3 (VSIG-3) or brain and testis-specific immunoglobulin superfamily (BT-IgSF), was originally identified as a member of the immunoglobulin superfamily (16, 17), and has been revealed to be expressed predominantly in the brain and testis in mammals (17). Latest studies showed that IgSF11 regulates synaptic transmission and plasticity by the interaction with the postsynaptic scaffolding protein PSD-95 and AMPA glutamate receptors (AMPARs) (18). Furthermore, IgSF11 has been identified as a ligand of the V-domain Ig suppressor of T-cell activation (VISTA), also known as PD-1H (19, 20), and which was revealed to have inhibitory effects on T cell functions (21, 22). However, Watanabe et al. demonstrated that IgSF11 expression has been characterized to be upregulated in colorectal cancers and hepatocellular carcinomas as well as intestinal-type gastric cancers (23). A recent study revealed that the binding interaction of IgSF11 to VISTA on activated T cells inhibits T-cell proliferation as well as cytokine and chemokine production (21).

In this study, we described an interestingly elevated expression profile of IgSF11 in high versus low human glioma patients. *IgSF11* gene appeared to be one of the most highly expressed immune checkpoints in the human glioma tumor microenvironment, compared to *PDL-1*, *Gal-9*, and *CD155*. IgSF11 protein was also revealed in various glioma patients’ samples. Interestingly, IgSF11 protein appeared to show a similar expression profile on different glioma grades, suggesting that *IgSF11* gene might be subject to post-transcriptional regulation. This regulation could also be linked to the essential role that the protein plays in the brain homeostasis, regulating synaptic transmission and plasticity (18). Finally, elevated *IgSF11* transcript expression correlated to a poor clinical outcome. Altogether, our data indicate that IgSF11 could be considered as a promising therapeutic target in advanced gliomas.

## Materials and Methods

### Patients and Samples

Total mRNA expression was assessed in a total of 52 specimens of glioma samples. Twenty glioma tissues at different grades: 10 specimens of high grade glioma (9 Glioblastomas grade IV, 1 Ependymoma grade III) and 10 of low grade (seven astrocytomas grade I, two oligodendrogliomas grade II, and one Ependymoma grade II); 22 peripheral blood mononuclear cell (PBMC) samples taken before surgery and 10 taken after surgery at the Ibn Rochd University Hospital, neurosurgery department (Casablanca, Morocco). As for the control, 10 specimens of PBMCs were taken from healthy donors, at the regional blood transfusion Center (Casablanca, Morocco). Patients had been previously diagnosed with glioma. Selected patients had not undergone any therapy before tumor resection. Glioma samples were classified according to the World Health Organization (WHO). Clinical information was obtained from the medical records of the patients.

### TCGA Data Analysis

Transcriptome data of 667 glioma patients were collected from The Cancer Genome Atlas (TCGA) (http://cancergenome.nih.gov/). During the analysis with TCGA RNAseq data, expression values were log converted. All data analysis and statistical tests were independently performed by two different people in the lab.

### Peripheral Blood Mononuclear Cell Isolation

Peripheral Blood Mononuclear Cells (PBMC) were isolated by density gradient centrifugation as described in the manufacturer’s protocol. A 5 ml of human peripheral blood was first mixed with 5 ml of saline solution (0.9% NaCl) which was added to 5 ml of lymphosep, lymphocyte separation medium (Biowest, France). The total was then centrifugated at 350 g for 10 min. The layer corresponding to the PBMCs was collected and then washed twice in 0.9% NaCl.

### RNA Isolation and Reverse Transcription (RT)

Total RNA was extracted from PBMCs and frozen glioma samples using TRIzol reagent (Invitrogen, France) as previously described (24). RNA concentration and quality were measured using the NanoVueTM Plus Spectrophotometer (GE Healthcare, UK), then cDNA was synthesized using Tetro Reverse Transcriptase Enzyme (Bioline, France) from 0.5 μg of total RNA in a 20 μl of reaction mixture according to the manufacturer’s instructions, mixed with 1 μl of Random Hexamer Primer 25µg (Bioline, France) and 4 μl of RNase-Free water, then incubated at 70°C for 5 min to break the secondary structures of RNA.

Next, 4 μl of Tetro Reverse Transcriptase buffer, 4 μl of dNTP (10 mM), 0.5 μl of RNase Inhibitor (Invitrogen, France), 0.5 μl of Tetro Reverse Transcriptase Enzyme (Bioline, France), and 1 μl of RNase-Free water were added and incubated at 25°C for 10 min then at 45°C for 30 min then at 85°C for 5 min.

### Real-Time RT-PCR

Gene expression was performed by real-time PCR in the presence of the fluorescent dye SYBR ™ Green PCR Master Mix (Thermo Fischer). *β*-actin was used as a housekeeping gene to analyze relative expression of *IgSF11*. Experiments were executed in a 20 μL reaction volume with specific primer pairs used at 10 µM for all genes.

PCR was programmed as follows:

10 min at 95°C for polymerase activation and sample denaturation, then 40 cycles of 15 s at 95°C and 60 s at 60°C for annealing and extension. Fluorescence readings at the end of the extension phase of each cycle were used to estimate the values for the threshold cycles (Ct). The Ct values for each gene were converted into relative quantification (2^-ΔCt^).

Primer pairs:

***β*-actin** Forward: 5’-TGGAATCCTGTGGCATCCATGAAAC-3’

Reverse: 5’-TAAAACGCAGCTCAGTAACAGTCCG-3’

***IgSF11*** Forward: 5’-GGCATTCCTCGACCAACTTA-3’

Reverse: 5’-ATTAGAAGCCACGCACTGGT-3’

### Immunohistochemistry (IHC)

Thirty paraffin-embedded human glioma tissues (13 low grade and 17 high grade cases) were sectioned (thickness of 3–4 µm). First, samples were incubated at 65˚C for 1 hour then at 37°C overnight before being deparaffinized and rehydrated. For antigen retrieval step, the water bath method was performed, using PT Link (Dako, Denmark) and a high pH (pH = 9) retrieval solution (EnVision Flex target retrieval solution high PH (x50) 30 ml, Dako, Denmark) at 98°C for 20 min.

To block the endogenous peroxidase activity, samples were immersed in 3% hydrogen peroxide (EnVision flex peroxidase-blocking reagent, Dako, Denmark) for 10 min at room temperature, followed by incubation in wash buffer (EnVision flex wash buffer, Dako, Denmark) two times for 2 min each to reduce non-specific binding. Slides were then incubated with a primary monoclonal mouse anti-human IgSF11 antibody at (1:150) (LifeSpan BioSciences, Seattle, United States). For each case, a second slide was used as a negative control with mouse IgG1 isotype control at 1: 200 dilution (LifeSpan BioSciences, Seattle, United States) at room temperature for 45 min.

After rinsing in wash buffer twice for 2 min each, slides were incubated with a secondary horseradish peroxidase-conjugated goat anti-rabbit anti-mouse IgG (EnVision Flex/HRP, Dako, USA) for 20 min at room temperature. Next, Slides were rinsed thoroughly in wash buffer, twice for 2 min each, prior to incubation with diaminobenzidine solution (EnVision DAB + CHROMOGEN, Dako, USA) to develop color for 10 min at room temperature. Finally, slides were counterstained with hematoxylin solution at room temperature for 1 min, dehydrated, and mounted to being examined under an Olympus light microscope (Olympus, Tokyo, Japan).

### Statistical Analysis

Statistical analysis was performed using GraphPad Prism 6.0 software (GraphPad Software, Inc., La Jolla, CA, USA). Statistical significance between mean values was determined by using Student’s t-test and one-way ANOVA. For survival curves, the Kaplan-Meier method was used based on log-rank test.

## Results

### *IgSF11* Gene Expression Is Upregulated in High Grade Glioma Tissues

In total, 20 glioma tissues (11 men and 9 women) were recruited in the current study. The characteristics of the enrolled patients were described in **Table 1**. Glioma patients were classified according to the WHO as follows: nine glioblastomas of grade IV, one ependymoma of grade III, seven astrocytomas of grade I, two oligodendrogliomas of grade II, and one ependymoma of grade II (**Table 1**).

**Table 1:** Characteristics of glioma patients.

Variable	Cases (%) (n = 20)
**Sex**	
• Male • Female	11 (55) 9 (45)
**Age**	
• ≤29 years • 30–50 years • ≤50 years	9 (45) 8 (40) 3 (15)
**WHO grade**	
• Low grade (I-II) • High grade (III-IV)	10 (50) 10 (50)
**Histological type**	
• Astrocytomas • Oligodendrogliomas • Ependymomas	16 (80) 2 (10) 2 (10)
**Smoking status**	
• Yes • No	5 (25) 15 (75)

**Figure 1:**
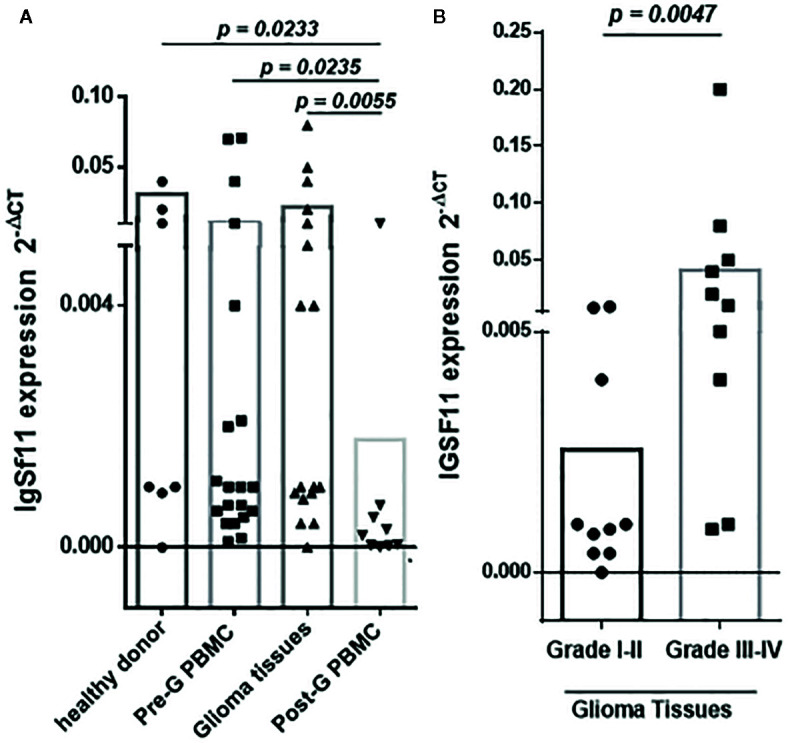
*IgSF11* gene expression is upregulated in high grade glioma tissues. *IgSF11* expression was assessed using RT-PCR analysis. **(A)** Higher expression of *IgSF11* gene in healthy donors, glioma tissues, and PBMC of the same patients before surgery (Pre-G-PBMC) compared to PBMC after surgery (Post-G PBMC). **(B)** Elevated expression of IgSF11 in advanced glioma grade (grade III-IV).

To assess the association between IgSF11 gene expression and glioma pathogenesis, 52 samples including 20 glioma samples and 32 PBMC specimens of the same patients (22 PBMCs before and 10 after surgery) and 10 healthy donors were analyzed. mRNA expression Levels of IgSF11 were evaluated by real time RT-PCR. *IgSF11* showed a significant mRNA expression in samples from glioma patients (**Figure 1A**). This expression was, however, similar to that observed in PBMCs from glioma patients taken before surgery. IgSF11 mRNA expression was also similar to that of PBMCs taken from healthy donors (**Figure 1A**). In addition, *IgSF11* transcripts were elevated in healthy donors, glioma tissues and PBMCs before surgery compared to PBMCs taken from patients after surgery (*p* = 0.0233), (*p* = 0.0055), and (*p* = 0.0235), respectively (**Figure 1A**). On the other hand, when we compared IgSF11 expression according to patients’ grades, we revealed a significantly higher expression in high grade glioma tissues relative to low grades (*p* = *0.*0047) (**Figure 1B**). Overall, these observations indicated that *IgSF11* mRNA was highly expressed in high grade gliomas compared to low grades.

**Figure 2:**
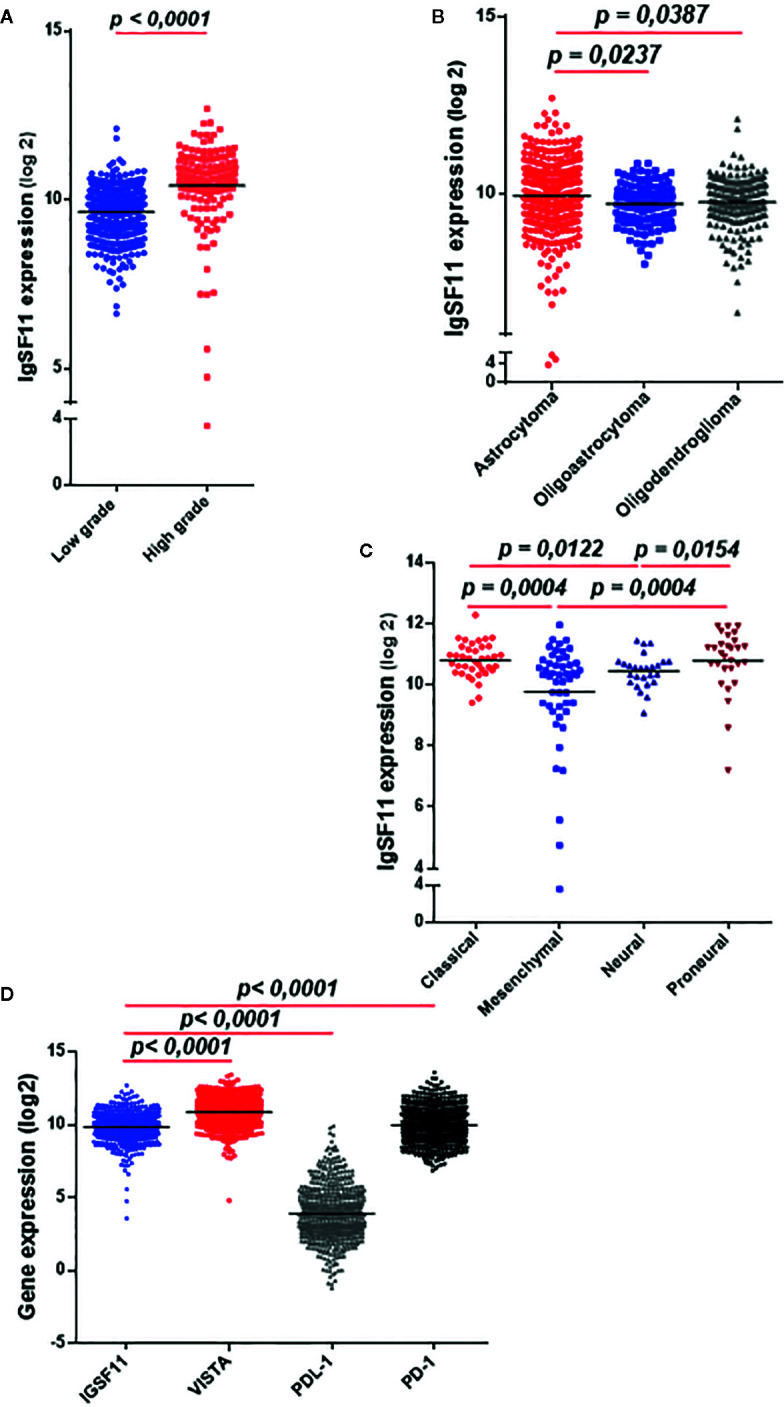
IgSF11 transcripts strongly expressed in high grade glioma in the TCGA cohort. RNAseq of glioma patients (n = 667) were evaluated using TCGA database. **(A)** *IgSF11* gene showed strong expression levels in high grade gliomas. **(B)** Astrocytoma exhibited high expression levels of *IgSF11* compared to Oligodendroglioma and Oligoastrocytoma. **(C)** Classical and Proneural glioma molecular subtypes showed high *IgSF11* expression in comparison with mesenchymal and neural. **(D)** *IgSF11* mRNA expression showed high expression levels compared with *PDL-1*.

In order to further strengthen the *IgSF11* mRNA expression results (which have been obtained with the Moroccan cohort) we evaluated *IgSF11* expression in a separate cohort. We assessed RNA-sequencing data of 667 gliomas patients from the TCGA database. Samples were analyzed and graded according to the WHO classification (**Table 2**). The expression profile of *IgSF*11 gene was significantly linked to glioma grades (*p* < 0.0001), histological type (*p* = 0.0169), patients age (*p* < 0.0001), molecular subtype (*p* = 0.0001) and IDH mutation status (*p* = 0.0018). High grade gliomas (glioblastoma) presented high level of *IgSF11* expression compared to low grade (*p* < 0.0001) (**Figure 2A**). In addition, astrocytoma showed an elevated expression of *IgSF11* in comparison to oligoastrocytoma (*p* = 0.0237) and oligodendroglioma (*p* = 0.0387) (**Figure 2B**). Further analysis exhibited an elevated expression of *IgSF11* in classical and proneural molecular subtypes, in comparison to neural (*p* = 0.0122 and *p* = 0.0154, respectively) and mesenchymal (*p* = 0.0004) (**Figure 2C**). Interestingly, *IgSF11* expression level appeared to be higher in all glioma samples (high and low grades) compared to *PDL-1* (*p* < 0.0001) (**Figure 2D**). IgSF11 protein was also detected in human gliomas.

**Table 2:** Expression of *IgSF11* according to glioma patient characteristics in the TCGA cohort.

Variable	Cases (%) n	*p* value
**Sex**		
• Male • Female	381 (57.20) 285 (42.8)	0.5791
**Age**		
• ≤29 years • 30–50 years • ≤50 years	79 (11.86) 284 (42.64) 303 (45.5)	<0.0001
**WHO grade**		
• Low grade (II-III) • High grade (IV)	514 (77.17) 152 (22.83)	<0.0001
**Histological type**		
• Astrocytoma • Oligoastrocytoma • Oligodendroglioma	346 (51.87) 130 (19.49) 191 (28.63)	0.0169
**Glioblastoma subtype**		
• Mesenchymal • Classical • Neural • Proneural	49 (34.26) 39 (27.27) 26 (18.18) 29 (20.28)	0.0001
**IDH mutation status**		
• Yes • No	99 (36.13) 175 (83.87)	0.0018

**Figure 3:**
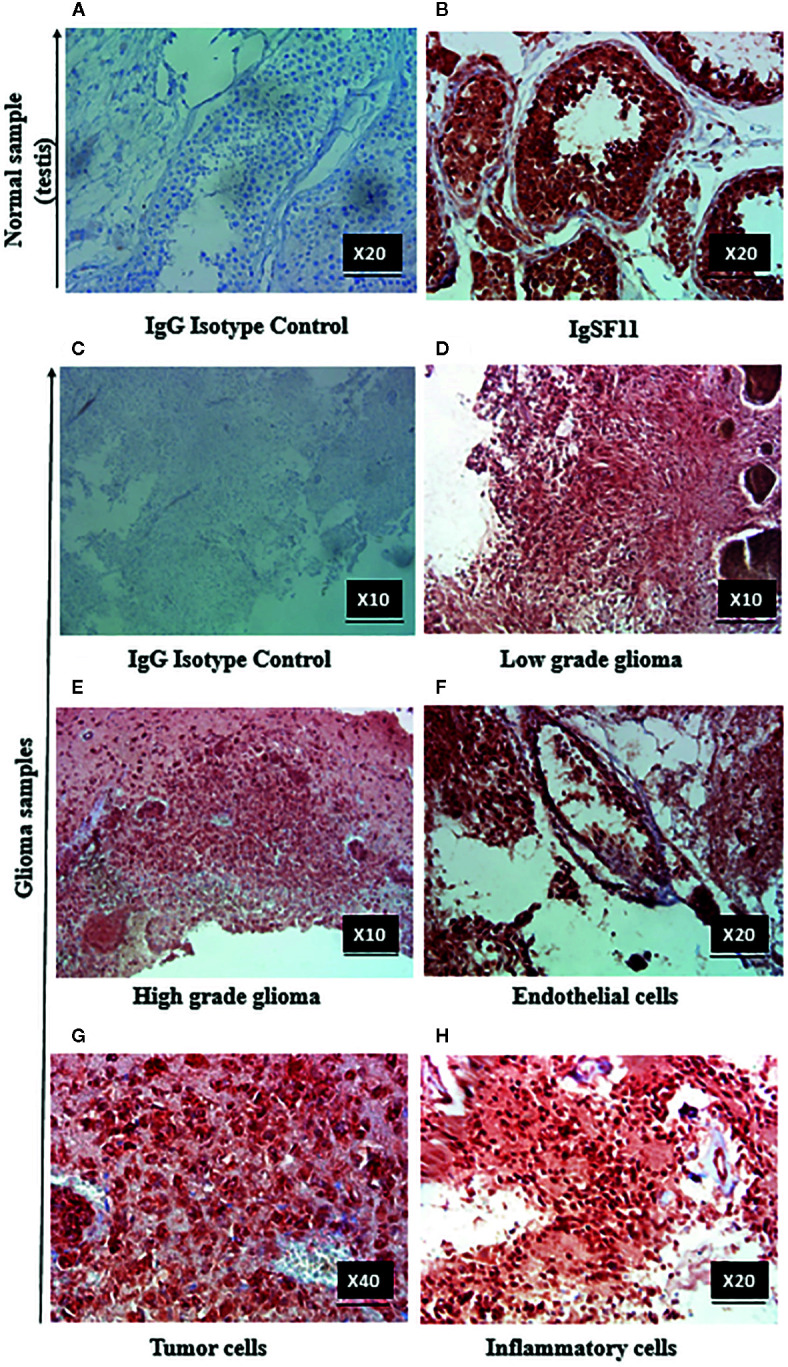
IgSF11 protein was detected in human gliomas. IgSF11 protein was detected in human glioma tissues using immunohistochemistry assay. **(A)** Negative control staining in normal testis tissue with mouse IgG1 isotype control (magnification x20). **(B)** Positive staining of IgSF11 in normal testis (magnification x20). **(C)** Negative control staining of glioma cases with mouse IgG1 isotype control (magnification x 10). **(D)** Positive staining of IgSF11 in low grade glioma (Astrocytoma I) (magnification x10). **(E)** Positive staining of IgSF11 in high grade glioma (Glioblastoma) (magnification x10). **(F)** Positive staining of IgSF11 on endothelial cells (magnification x 20). **(G)** Positive staining of IgSF11 on tumor cells (magnification x 40). **(H)** Positive staining of IgSF11 on inflammatory cells (magnification x 20).

In order to confirm IgSF11 gene expression results obtained at the transcript level (**Figure 1**), IgSF11 protein analysis was performed on 30 human glioma samples (13 low grade (I/II) and 17 high grade (III/IV) cases) by immunohistochemistry. When IgG1 isotype control was assessed in testis tissue (used as a positive control for IgSF11 protein expression) (**Figure 3A**), no staining was detected. However, staining of IgSF11 was observed with the same tissue when a specific anti-IgSF11 mAb was used in the same conditions **Figure 3B**). IgG1 isotype was used as a negative control to assess IgSF11 protein expression in glioma tissues (**Figure 3C**). Surprisingly, strong IgSF11 staining was observed in all cases of glioma (low and high grades) (**Figures 3D, E**), contrasting with mRNA data (**Figure 1B**). This suggests that IgSF11 might be subject to a post-transcriptional regulation. IgSF11 protein was detected in endothelial cells (**Figure 3F**) and tumor cells (**Figure 3G**) in all grades of gliomas. Besides, about 16.66% of glioma samples (5 out of 30 cases) showed a positive staining of IgSF11 on tumor-associated inflammatory cells (**Figures 3A, H**). Overall, our data indicated that IgSF11 protein was strongly expressed on tumor and inflammatory cells in human gliomas.

### *IgSF11* Transcripts Positively Correlated With Other Critical Immune Checkpoints

Using the same cohort of patients (TCGA), we assessed the correlation of *IgSF11* expression with three critical immune checkpoints, *PDL-1 and PD-1* (both known to present high levels of expression in advanced glioma grades) (23–25) and VISTA, which is known as IgSF11 receptor (20).

**Figure 4:**
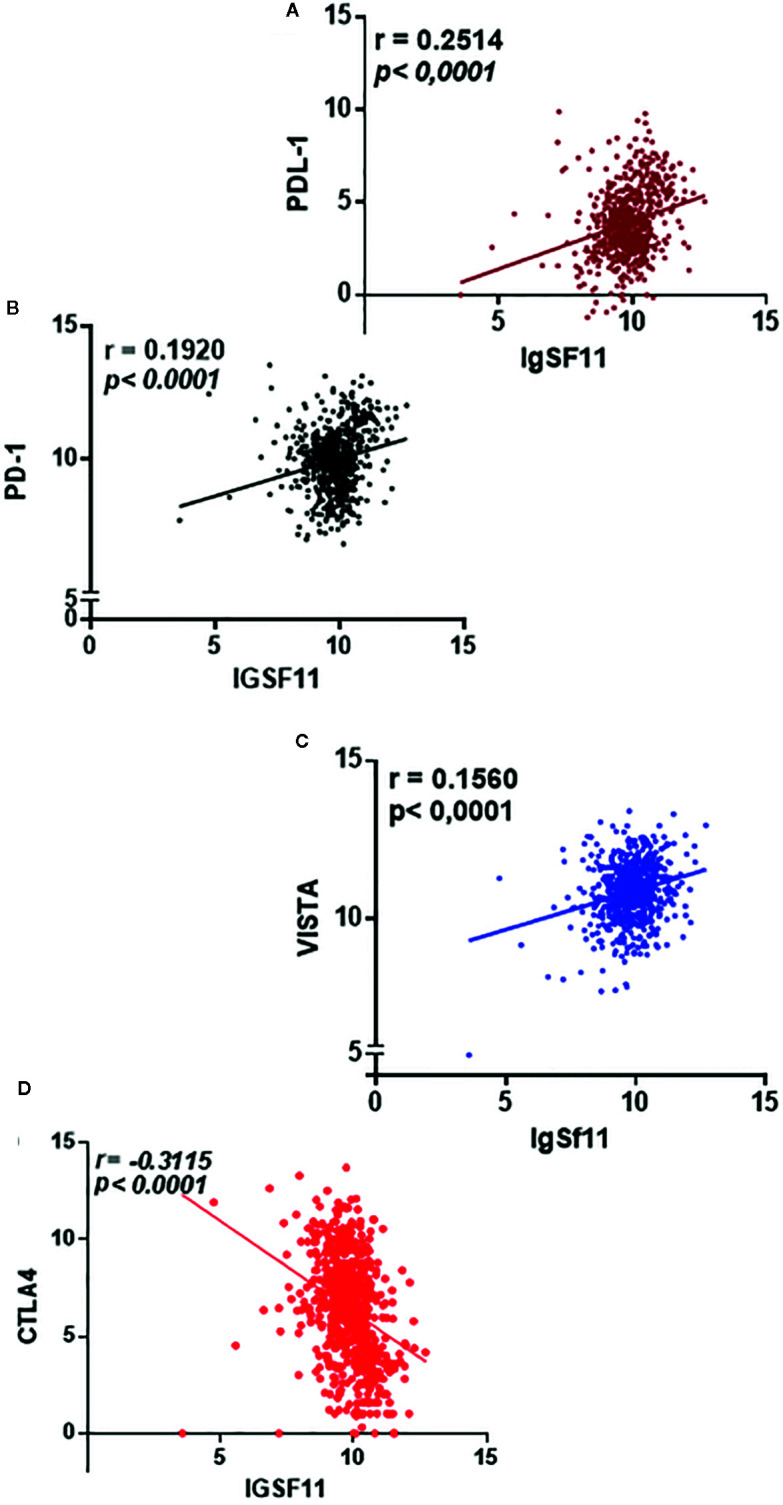
IgSF11 expression positively correlated with critical immune checkpoint regulators. **(A)** IgSF11 presented positive correlation with *PDL-1.* **(B)** *IgSF11* positively correlated with *PD-1*. **(C)** IgSF11 positively correlated with *VISTA*. **(D)** *IgSF11* negatively correlated with CTLA-4.

Interestingly, *IgSF11* was positively correlated with *PDL-1* (*p* < 0.0001, r = 0.2514) (**Figure 4A**), *PD-1* (*p* < 0.0001, r = 0.1920) (**Figure 4B**), *VISTA* (*p* < 0.0001, r = 0.1560) (**Figure 4C**) and negatively correlated with *CTLA-4* (*p* < 0.0001, r = -0.3115) (**Figure 4D**) (confirming that *IgSF11* expression correlated with that of its receptor, VISTA. Together, these observations suggest that tumor cells may use *IgSF11* in addition to other previously reported immune checkpoints, such as PD-1 and PDL-1 axis, to institute an immune suppressed microenvironment.

### Significant Immune Cell Infiltration but Strong Immuno-Suppressive Microenvironment in Patients With High Levels of IgSF11

**Figure 5:**
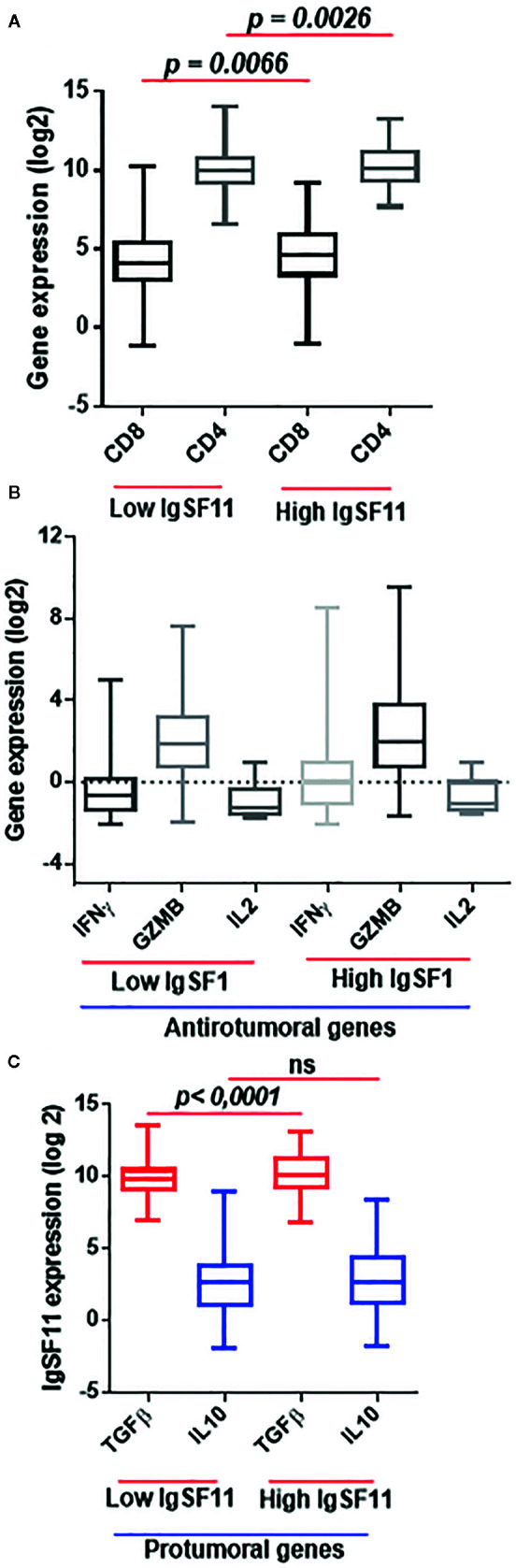
High expression of *IgSF11* transcripts correlated to an immunosuppressive microenvironment of glioma patients. **(A)** *CD4* and *CD8* gene expression were elevated in high *IgSF11* expression. **(B)** Anti-tumoral genes (*IFNγ*, *Granzyme B*, *IL-2*) exhibited no significant difference in high versus low *IgSF11* expression profile. **(C)** *TGFβ* showed strong expression levels in glioma patients with high *IgSF11* expression.

To explore the association between *IgSF11* expression and the presence of various immune cell populations within glioma microenvironment, we performed a duple clustering of glioma cases using the median as a cut off for patient’s stratification. The first set with high *IgSF11* expression, and a second one with lower expression. Thereafter, we evaluated CD4 and CD8 mRNA expression. Patients with high *IgSF11* expression presented high levels of expression of *CD4* and *CD8* compared to low IgSF11 expression (*p* < 0.0001) (**Figure 5A**), suggesting that although glioma microenvironment is highly infiltrated by *CD8* and *CD4* cells, the latter would be able to exhibit limited effector functions owing to the high expression of *IgSF11* gene, in addition to other immune suppressive genes. As for T lymphocyte-related cytokines, we analyzed gene expression of two separate sets, *TGF-β* and *IL-10*, which are involved in the process of strongly inhibiting both CD8 and CD4 T cell functions. On the other side, we investigated a set of genes, including *IL-2*, *IFNγ*, and *Granzyme B*, whose upregulation is rather associated to an efficient anti-tumoral immune response. Interestingly, the expression of pro-inflammatory genes (*IL-2*, *IFNγ*, and *Granzyme B*) did not show a significant difference between the two groups (high versus low IgSF11 expression profile (**Figure 5B**).

In contrast, the immunosuppressive gene *TGF-β* exhibited high levels of expression in glioma cases with higher levels of *IgSF11* gene expression (*p* < 0.0001) (**Figure 5C**). Together, these observations indicated that patients with high level of *IgSF11* expression, would likely exhibit significant CD4 and CD8 cell infiltration, which would be, however, weakly functional, due to a strong immuno-suppressive microenvironment, which is known to negatively impact the patient’s clinical outcome.

### Elevated Expression of *IgSF11* in Glioma Patient’s Microenvironment Associated to a Poor Overall Survival

**Figure 6:**
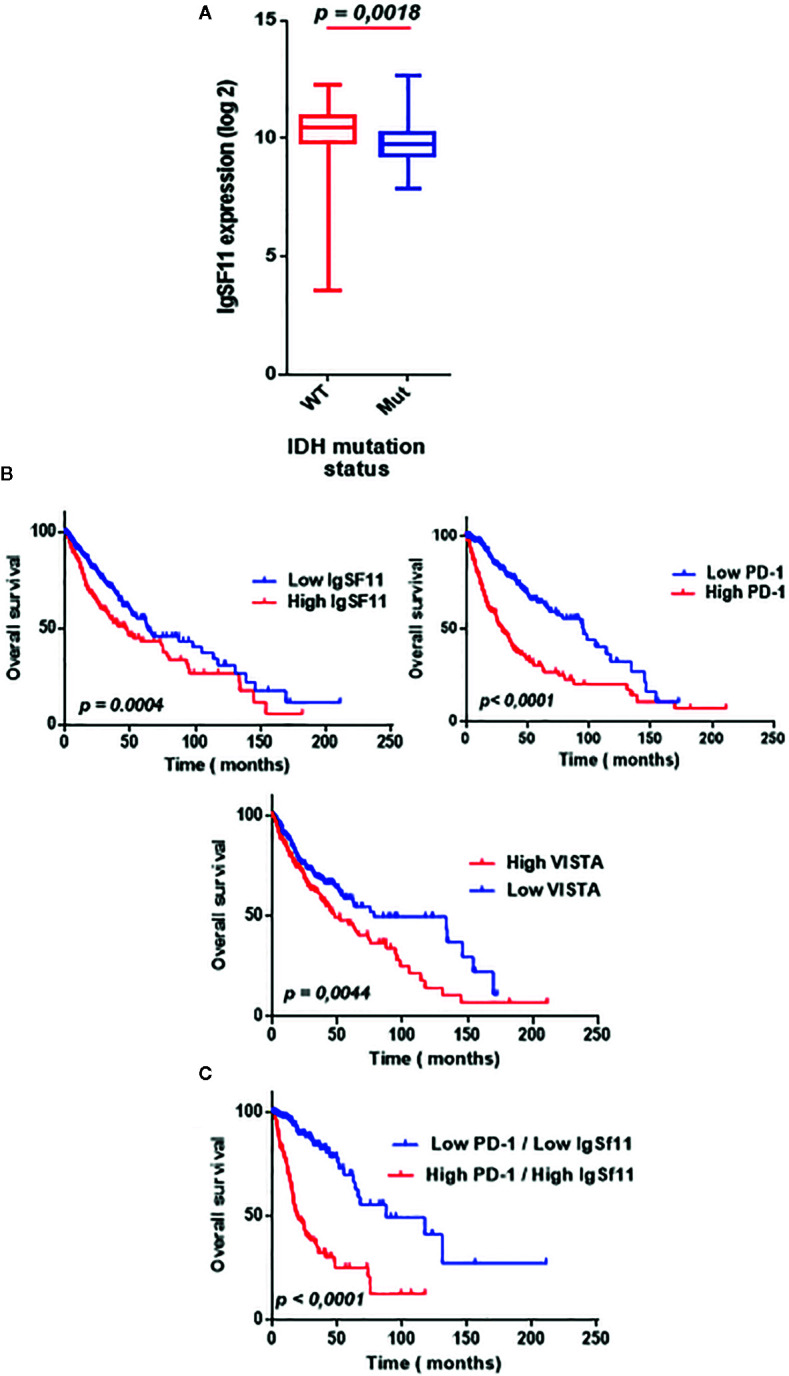
Elevated expression of IgSF11 in glioma patients associated to a poor overall survival. **(A)** *IgSF11* upregulated in glioma patients with *IDHwt* compared with *IDHmut* status. **(B)** High *IgSF11, PD-1 and VISTA* expression levels associated to a poor overall survival. **(C)** Elevated expression of both *IgSF11* and *PD-1* in the same glioma patients correlated to a poorer overall survival.

To clarify the relationship between *IDH* mutation status and *IgSF11* expression, transcriptomic data of patients with *IDH* mutation status was analyzed. *IDH* wild-type glioma showed a significantly distinct pattern of *IgSF11* expression from *IDH* mutant glioma with significant upregulation in *IDH* wild-type glioma (*p* < 0.0001) (**Figure 6A**). Furthermore, to explore the impact on survival, we examined the prognostic value of *IgSF11*. As demonstrated, using Kaplan-Meier curves, glioma patients with lower *IgSF11* expression had prolonged survival in comparison to patients with higher expression (*p* = 0.0004). When *PD-1* and *VISTA* genes, were assessed, the results showed that glioma patients who expressed high levels of *PD-1* and high levels of *VISTA* presented a poorer survival compared to those who expressed lower levels (*p* < 0.0001) (*p* = 0.0078) (**Figure 6B**). Remarkably, patients who presented high expression levels of both *IgSF11* and *PD-1* showed a worse survival outcome compared to those with low expression of both genes (p<0.0001) (**Figure 6C**). These results indicated that *IgSF11* could be considered as a negative prognostic marker in gliomas and that dual blocking of PD-1/IgSF11 pathways could be considered as promising future combined therapy for glioma patients.

## Discussion

Immune checkpoint inhibitors blockade is one of the most promising approaches to enhance anti-tumor immune response and it has made great stride in mending patient’s clinical outcome for multiple cancer types (26, 27). Gliomas are known to be the most frequent and fatal brain tumors in adults (28). As far as here, despite treatment of glioblastoma patients with conventional therapies, the prognosis is still middling (29). Despite the hope that immunotherapy has brought in the field of cancer treatment (30), the majority of glioma patients did not respond to the blockade of usual immune checkpoints pathways (28–31). This has catalyzed our interest in exploring additional targets, including the recently discovered one, IgSF11 (23). Thus, the main objective of this study was to explore the role of IgSF11 in human gliomas.

Our study demonstrated that: 1) *IgSF11* gene expression was elevated in high grade glioma patients, 2) IgSF11 protein was also detected in different glioma patients, 3) The high *IgSF11* transcript expression in high grade gliomas was corroborated in an independent cohort, TCGA, 4) *IgSF11* transcript levels positively correlated with other critical immune checkpoints, 5) Patients with elevated IgSF11 levels, exhibit immune cell infiltration but an immuno-suppressive microenvironment, and 6) Elevated expression of *IgSF11* in glioma patients associated to a poor overall survival.

At the best of our knowledge, this is the first investigation of the role of *IgSF11* in clinically resected human glioma tumors. Also, this is the largest and most comprehensive study describing the expression of IgSF11 in human glioma samples using two distinct cohorts.

IgSF11 expression has been evaluated on other cancer types, such as gastrointestinal and hepatocellular carcinoma (21, 32). Recently, it has been reported that IgSF11 has an inhibitory effect on T cell function (21). In these studies that are compatible with the present investigation, it has also been demonstrated that IgSF11 expression is upregulated in these types of cancer. In addition, unlike our transcriptomic data, IgSF11 protein expression in the tumor microenvironment was not found to be associated with the grade nor the histological type of gliomas. Considering its physiological role in the brain, which is the regulation of synaptic transmission and plasticity (18). In our study, IgSF11 protein was demonstrated to be expressed on tumor samples of all grades and also on tumor-associated inflammatory cells (in 5 out of 30 cases), which suggests that IgSf11 may play a double role, as a receptor, on tumor microenvironment-infiltrating inflammatory cells, and as a ligand, on glioma cells, in order to enhance the immuno-suppressive action upon its interaction with VISTA, which has been shown previously by Ghouzlani et al. **(in revision)** that it is expressed on both. (Ghouzlani et al., **in revision)**

This is the first report to the best of our knowledge on the role of IgSF11 in human glioma progression. In the present study, *IgSF11* expression was significantly and positively correlated with that of *PDL-1*, *PD-1*,and *VISTA* and negatively with that of *CTLA-4*.

This suggests that IgSF11 would use similar mechanisms as other critical immune checkpoints such as the *PDL-1*/*PD-1* axis, whose activity is known to be associated with a bad prognosis for glioma patients. On the other hand, it was reported that IgSF11 is a ligand of VISTA (21), and that IgSF11-VISTA interaction results in the suppression of T cell activity. This has prompted us to explore the role of IgSF11 in glioma progression using two independent tumor cohorts, local human glioma samples, and TCGA cohort. One main observation of this study is that *IgSf11* and *VISTA* expression correlated positively and significantly. This is an important observation since the combined blocking of the IgSF1-VISTA pathways, along with other immune checkpoints could be of interest in glioma treatment. Chraa et al. reported that the glioma microenvironment is infiltrated by several subpopulations of T lymphocytes that could impact cancer progression (33). Our study revealed that patients with high *IgSF11* expression profile would present high infiltration of CD4 and CD8 cells compared to patients with low *IgSF11* expression. However, glioma microenvironment infiltrating CD8 and CD4 cells would exhibit limited effector functions, owing to the higher expression level of the immuno-suppressive molecule, IgSF11, in addition to other immune checkpoints. As for T lymphocyte-related cytokines, the expression of pro-inflammatory genes (*IL-2*, *IFNγ*, and *Granzyme B*) did not show a significant difference between the two groups (high versus low IgSf11 expression profile), even though T cell infiltration seems to be significantly higher in the group of patients with elevated expression of IgSf11. If these observations are confirmed, this would mean that these pro-inflammatory/anti-tumoral cytokines are likely to be silenced due to the highly immuno-suppressive microenvironment. Indeed, the potent immunosuppressive cytokine *TGF-β*, which is known to be involved in the process of glioma progression (34), showed high levels of expression in patients with high levels of *IgSF11*.

Remarkably, high expression levels of *IgSF11* were associated with worse patient survival. Also, glioma patients exhibited elevated expression of *PD-1* or *VISTA* showed a bad overall survival. Interestingly, patients presenting high expression levels of both *IgSF11* and *PD-1* showed even weaker overall survival. These results indicated that *IgSF11* could be considered as a negative prognostic marker in glioma and *PDL-1/IgSF11* blockade could be also considered as a very promising combined therapy for advanced glioma.

In summary, in this study, which corresponds to the first evaluation of *IgSF11* expression and role in human glioma, we presented evidence for gradual expression of IgSF11 in patients presenting with gliomas, depending on distinct grades. High IgSF11 expression in advanced glioma patients correlated with higher infiltration of immune cells, which would be weakly functional, due to the highly immuno-suppressive microenvironment (elevated expression of immune checkpoints and cytokines such as TGFβ). Our study identified IgSF11 as a possible promising therapeutic target in advanced human glioma.

## Data Availability Statement

The datasets presented in this study can be found in online repositories. The names of the repository/repositories and accession number(s) can be found in the article/supplementary material.

## Ethics Statement

The studies involving human participants were reviewed and approved by The Ethical Board of the Ibn Rochd University Hospital of Casablanca. Written informed consent to participate in this study was provided by the participants’ legal guardian/next of kin.

## Author Contributions

AG collected, analyzed, and interpreted data, and wrote the manuscript. SR collected and analyzed data. MK analyzed data. AL collected and analyzed data. AB designed research, analyzed and interpreted data, wrote the manuscript, and supervised the study. All authors contributed to the article and approved the submitted version.

## Funding 

This work was supported by the Moroccan Ministry of Higher Education and Research and The National Center for Scientific and Technical Research (CNRST) through a “PPR1” project coordinated by AB. AG was supported by a “CNRST” fellowship.

## Conflict of Interest

The authors declare that the research was conducted in the absence of any commercial or financial relationships that could be construed as a potential conflict of interest.

## Abbreviations

WHO, World Health Organization; GBM, Glioblastoma; IgSf11, Immunoglobulin superfamily 11 gene; PD-1, Programmed cell death 1; VISTA, V-domain Immunoglobulin suppressor of T cell activation; PDL-1, Programmed death-ligand 1; CTLA-4, Cytotoxic T lymphocyte antigen-4; PCR, Polymerase chain reaction; IL-2, Interleukin*-*2; IL-10, Interleukin*-*10; TGFβ, Transforming growth factor β; IFNγ, Interferon gamma; IDH, Isocitrate dehydrogenase; TCGA, The Cancer Genome Atlas; PBMC, Peripheral blood mononuclear cell; mRNA, Messenger RNA.
